# An examination of anxiety levels of nursing students caring for patients in terminal period

**DOI:** 10.12669/pjms.341.14285

**Published:** 2018

**Authors:** Behire Sancar, Ayse Saba Yalcin, Inci Acikgoz

**Affiliations:** 1 Dr. Behire Sancar, RN, PhD. Assistant Professor, Toros University, School of Nursing, Mersin, Turkey; 2 Dr. Ayse Saba Yalcin, PhD. Ankara University, Faculty of Health Sciences, Nursing Department, Ankara, Turkey; 3 Dr. Inci Acikgoz, PhD. Ankara University, Faculty of Health Sciences, Nursing Department, Ankara, Turkey

**Keywords:** Nursing, Student, Terminal Period, Anxiety, Provide Care

## Abstract

**Objective:**

To investigate the anxiety levels of the nursing students who are caring for the patients in the terminal period and to determine whether there is a difference between 3^rd^, 4^th^ grade in this direction.

**Methods:**

A 40-item “State and Trait Anxiety Scale” was used together with the questionnaire on “Determining the Level of Anxiety Levels of Nursing Students Caring for the Patient at the Terminal Period” for determining the data.

**Results:**

The mean scores and standard deviations of all students from the state and trait anxiety scales were respectively 41.95±5.06, 48.15±5.44. Averages of 3^rd^ state anxiety scale score was 42.03 ± 5.26, trait anxiety scale averages were 48.08 ± 5.59; Averages of 4^th^ state anxiety scale score was 41.85 ± 4.83, trait anxiety scale averages were 48.24 ± 5.30.

**Conclusion:**

In our study, it was found that there wasn't significant difference between the 3^rd^, 4^th^ grade students related to ill patient care in terms of high level of state and trait anxiety during communication and patient care. The state and trait anxiety scores of the students in both grades were found to be higher than the average scores of the scale's previous applications.

## INTRODUCTION

We all know death is inevitable but how and when it will be experienced is not known.^1^ Unexpected sudden deaths can occur as well as pre-death periods that last longer in the final stages of diseases or during old age. This process is called the “terminal stage”. General problems during this terminal stage vary according to the stage the patient is in, the interpretation of death and the support systems. These problems can be summarized as not being interested in the outside world, fear-related anxiety, excessive dependence, rebelliousness, attitudes and behaviors that will adversely affect health, tendency to be alone and showing angry behavior towards healthy people.^2^ It is very important to keep the life quality at the highest possible level during the terminal stage.^3^

Behavioral disorders and mental problems are two major problems in patients at the terminal stage.^4^ Some patients at the final stage of the disease may want to accelerate their death due to depression and despair. Interventions targeting depression and despair, social support and effective communication are important components of palliative care regarding the wish for a quick death.^5^

Palliative care is defined as an approach that improves life quality by early detection of pain, psycho-social and spiritual problems, and the avoidance of suffering in situations that threaten the life of the patient and the family.^6^,^7^ Patients at the terminal stage are separated from classic intensive care and hospital services and transferred to special care centers in many countries. This practice has been combined with home care implementation and forms the concept of “hospice”.^8^ The palliative care specialization in the UK has been approved in the US as a hospice (home-like institution providing such care to the patient) and “palliative medicine” specialty in the USA. Palliative care is an effective approach increasing the life quality of patients at the terminal stage.^9^ Palliative care services in Turkey are planned to be performed with the participation of family physicians.^10^ Nurses who are more involved with patients in this palliative care process have an important role.^11^

Nursing is a dynamic process that creates a nursing care plan under the light of the physical, emotional, mental and social health care needs of the individual; puts it into practice, and then evaluates it systematically.^12^ Today's nursing involves presenting the scientific knowledge related to humans and their lives to the individual and society using holistic skills.^13^ The effective communication established by the nurse with the patient at the terminal stage contributes to facilitating pain control and increasing the well-being and life quality of the patient.^14^

Most healthcare employees avoid working in clinics where fatal patients are found due to the fear of being inadequate and unsuccessful in these patients' care and they also develop some defenses such as appearing to be always busy.^15^

Anxiety, which is an important concept in explaining human behavior, is a psychological reaction to the excessive energy resulting from stress in an individual.^16^ These changes are caused by external and internal factors in the body. Individuals experience stress when they feel inadequate or experience very strong emotions. Stress is inevitable in students who perceive their own potential inadequacy and have difficulty in controlling their emotions when communicating with patients.^17^ Anxiety needs to be treated when it prevents the individual from enjoying life. In this regard, one should primarily focus on preventive measures.^18^ Nurses should therefore acquire general knowledge and skills regarding the care of the patients at the terminal stage during their training, before encountering such patients.^19^

Nursing students encounter patients at the terminal stage and provide nursing care to these patients during their training. This can be a cause of anxiety for student nurses at various levels. The extent of the anxiety experienced varies according to the personality characteristics of the student, the approach to the subject, and the readiness to provide care to these patients.

The purpose of this study was to investigate the anxiety levels of nursing students who provide care to patients at the terminal stage and to determine whether there was a difference between the nurses at various years of training.

## METHODS

The population of this descriptive type study consisted of the 3rd and 4th grade students of Ankara University Health Sciences Faculty of Health Sciences, Department of Nursing and the sample of 154 students who accepted to participate in the study and then answered the questions completely.

### Collection of the Data and the Ethical Aspects

“The Questionnaire Form for Identifying the Anxiety Levels of Students Providing Care to a Patient at the Terminal Stage” consisting of 25 questions prepared by the researchers and the “State Trait Anxiety Inventory” consisting of 40 items were used in obtaining the data. The institution and ethics committee permissions (dated 24 January 2013 –no. 662) necessary to conduct the study were obtained.

### State Trait Anxiety Inventory (STAI)

STAI was developed by Spielberger, Gorsuch and Lushene in 1970 and adapted to the Turkish culture by Oner and Le Compte (1983). It consists of 2 sub-scales with 20 expressions measuring state and trait anxiety. Emotions and behaviors specified in the State Anxiety Sub-scale are marked according to the degree of severity as 1- Never, 2- Somewhat, 3- A lot, 4- Completely; and Trait Anxiety Sub-scale items are marked as 1- Almost never, 2- Sometimes, 3- Mostly, 4- Almost Always. The conversion of the Turkish form of the State and Trait Anxiety Inventory was conducted with the two different techniques of experimental concept validity and criterion validity.^20^ There are two kinds of expressions in the scales: **(1)** direct and **(2)** reverse expressions.

Direct expressions state negative feelings while reverse expressions state positive feelings. There are ten reverse expressions in the state anxiety sub-scale, items no. 1, 2, 5, 8, 10, 11, 15, 16, 19 and 20. There are seven reverse expressions in the trait anxiety sub-scale, items no. 21, 26, 27, 30, 33, 36 and 39. A separate key is prepared for the direct and reverse expressions. The total weight of direct expressions is calculated with one key and the total weight of the reverse expressions with the other key. The total weighted score of the reverse expressions is subtracted from the total weighted score obtained for direct expressions. A previously fixed and constant value is added to this number. This constant value is 50 for the state anxiety sub-scale and 35 for the trait anxiety sub-scale. The final value obtained is the anxiety score of the individual. The scores obtained from both scales can theoretically vary between 20 and 80. The mean score of this scale as identified in the implementations has varied between 36 and 41.

### Analysis of the Data

The data were analyzed by using the SPSS 21.00 software program. The relationship between the State Anxiety Scale and Trait Anxiety Scale was investigated with correlation analysis; the relationship between the questionnaire form and the scale scores was analyzed with the t-test in independent groups and the relationship between the questionnaire form and the student years with the chi-square test. A p level <0.05 was accepted as significant in statistical evaluations. Only significant differences have been shown in the tables.

## RESULTS

The mean age and standard deviation for the students included in the study was 21.84±1.46. There were 144 females and 54.5% were 3rd year students. The majority of the students (70%) wanted to go to a clinic with a higher percentage of terminal stage patients during the distribution of the internship places at school. The ratio of those who felt providing care to patients at the terminal stage was necessary before starting the profession was 92%. Inadequacy was stated as the cause by 58% of those who did not find it necessary. The number of students who confessed to inadequate knowledge about the care of patients at the terminal stage at the beginning of their internship was 52%.

We found that 62% of the students had not been able to communicate with their patient when they first met. The opinion “Their state affects me too much, I feel very sad” was expressed by 59% of the nurses about the patients at the terminal stage as they had a short time to live and required the help of someone else until the end of their life.

Giving care to patients affected the mental health of 73% of the students negatively and 20% stated that they felt inadequate to provide care. “The patient being at the terminal stage, and knowing that the patient will not be able to recover despite all intervention and care” was stated as the main reason of feeling sad for the patient at the terminal stage by 73% of the students.

When asked about the most difficult care performed for the patients at the terminal stage, 40% of the students stated it was wiping the bottom of the patients. Although they knew that the patients would not recover, 72% of the students felt happy to be able to help these patients.

The content of the talk with patients at the terminal stage was determined together with the family by 52% of the students and they tried to communicate according to the patient's areas of interest. The percentage of students reporting that they did not want to work in departments containing patients at the terminal stage after graduation because they felt sad was 36%.

Table-I reveals no difference between the 3rd and 4th year students in terms of the scale scores (p>0.05). However, the mean state and trait anxiety scores of the students in both years were above the mean scores of the scale as determined in previous implementations. The trait anxiety of the students was also higher than their state anxiety.

**Table-I:** The state trait anxiety inventory means score and standard deviation of the students.

	Year	N	X̄	S
State	3	84	42.03	5.26
Anxiety score	4	70	41.85	4.83
	Total	154	41.95	5.06
Trait	3	84	48.08	5.59
Anxiety score	4	70	48.24	5.30
	Total	154	48.15	5.44

A positive correlation of 24% (weak) was obtained between the state anxiety scale and the trait anxiety scale (p<0.01). The trait anxiety of the students with high state anxiety was also high.

A statistically significant difference was found between the state of communicating when the patient is first met and the trait anxiety scale result (p <0.05). On the other hand, no significant difference was found between communicating when the patient is first met and the state anxiety scale result (p> 0.05).

A significant difference was found between being uncomfortable when asking questions regarding the course of the disease and the trait anxiety scale result (p <0.05). The mean trait anxiety score of those who experienced discomfort was 49.17 ± 5.71, and the mean trait anxiety score of those who did not experience discomfort was 46.84 ± 4.76. A significant difference was obtained between giving care to the patients at the terminal stage affecting the mental health negatively and the trait anxiety scale result (p <0.05). The mean trait anxiety scores were higher in those whose mental health was affected negatively.

A significant difference was found between being able to wish good things to the patient and the state anxiety scale result (p <0.05). The mean state anxiety score was higher in those who could wish good things.

A statistically significant difference between the 3rd and 4th years about the views of the students regarding the patients (p<0.05) is shown in Table-III. Although the mental health of the majority of the students was affected, the 4th grade students were affected more. This may be due to the higher incidence of giving care to patients during the 4th year. Very few (20%) of the students who wished good things to the patient were 4th year students. The 4th year students may not be able to wish good things because they know the situation of the patient better as they are about to graduate.

**Table-II:** The Comparison of the student views regarding the patients with the state/trait anxiety scale results (n: 154).

The Scales	n	X̄	S	t*	p
*Communicating when the patient is first met*					
*Trait Anxiety Scale*					
Yes	59	47.01	5.43	-2.218	0.028
No			95	48.96	5.34
*Being uncomfortable about asking questions regarding the course of the treatment*					
*Trait Anxiety Scale*					
Yes	91	49.06	5.66	2.675	0.008
No			63	46.70	4.76
*Negative effect on mental health of giving care to patients at the terminal stage*					
*Trait Anxiety Scale*					
Yes	112	48.88	5.52	2.767	0.006
No			42	46.21	4.79
*Being able to wish good things for the patients*					
*Trait Anxiety Scale*					
Yes	60	43.32	4.98	2.815	0.006
No			94	41.03	4.92

* The t-test in independent groups was used.

**Table-III:** The Comparison of the student view regarding the patients by year (n:154).

Years								

The Students Views Regarding The Patients	3		4		Total		Test*	p=

	n	%	n	%	n	%		
Requiring Help							χ^2^ = 18.256	sd=2,0.00
-Their state affects me significantly, I feel very sad.	62	73.8	29	41.4	91	59.1		
-My thoughts vary according to the age of the patient.	9	10.7	21	30.0	30	19.5		
-It is a difficult situation for the caregiver.	13	15.5	20	28.6	33	21.4		
Mental Health Being Affected Negatively							χ^2^ = 4.451	sd=1,0.035
Yes	55	66.3	57	80.3	112	72.7		
No	28	33.7	14	19.7	42	27.3		
Being Able to Wish Good Things							χ^2^ = 5.818	sd=1, 0.016
Yes	40	47.6	20	28.6	60	39.0		
No	44	52.4	50	71.4	94	61.0		
The Situation that makes one feel the Worst							χ^2^ = 8.341	sd=2,0.039
- The patient being at the final stage, knowing that the patient will not be able to get better despite all the interventions and care.	68	81.0	44	62.9	112	72.7		
-Thinking about what their relatives experience and will experience.	6	7.1	10	14.3	16	10.4		
- Fearing that I may experience the same things when I empathize.	10	11.9	16	22.9	26	16.9		

* The Chi-square test was used.

## DISCUSSION

The anxiety levels of the nursing students who provided care for patients at the terminal stage and the factors influencing them were investigated in this study. Almost all the students included in the study stated that they wanted to go to a clinic with terminal stage patients during their internship. Another study also found that the majority of the students wanted to provide care to a patient at the terminal stage and stated that nurses should use their time and energy for patients at the terminal stage.^21^

Almost all students included in this study thought it was necessary to provide care to these patients during their training; however, they experienced stress, concern and anxiety both during this care and in the long term. The high mean trait anxiety scores of the students who expressed that their mental health was affected negatively support this result.

The communication that they establish when they first encounter a patient at the terminal stage causes constant anxiety in the students. The majority of the students also had inadequate knowledge about the terminal stage. Another study concluded that the majority of nursing students found their knowledge about end-of-life care inadequate and more than half experienced anxiety while giving care to a dying patient.^22^ Our results were similar.

The mean state anxiety scores of the students “who could with good things despite knowing the course of the disease” were high in this study. Breitbart et al. found in their 2000 study that depression and despair contributed to the desire for rapid death in terminal stage cancer patients.^5^ The high state anxiety of the students who could wish good things and the anxiety they felt while talking with these patients who experience depression and despair can be explained with the fear of making a mistake and being unsuccessful in communicating.

The scores obtained by 3rd and 4th year nursing students from the trait anxiety scale were higher in both groups with no difference between the years in this regard. Another study reported that nursing students prioritized physical care when providing nursing care to patients at the terminal stage and additionally found communication and psychological support initiatives important.^19^ These results show that the students adopted a holistic care approach.

Nursing students therefore show responses indicating alarm in the form of fear, confusion, and panic as related to the stress they experience when providing physical and psychological care to the patients at the terminal stage. The anxiety can be controlled and self-confidence restored in those who are successful as a result of active or passive resistance behaviors but trait anxiety occurs in case of failure.

The anxiety and stress levels of the health team working with cancer patients were found to be different than the health team working with other patients and the most important stress factor to be the patients at terminals stage and their families in the study conducted by Bostancı et al.^23^ According to the results of another study, students experience feelings of sadness and fear when they encounter death or an individual who is about to die and feel helpless and inadequate while giving care to the individual who is about to die during their training.^24^

### Limitations of the Study

This study was performed on nursing students attending a single state university. The study was limited to 154 students of Ankara University's Department of Nursing.

## CONCLUSION

According to the findings of this study, the patient being at the terminal stage and knowing that the patient will not be able to recover despite all interventions and care increases the anxiety level of nursing students. The nursing students were found to experience anxiety during giving care to the patients at the terminal stage and for a long time afterwards. Including subjects related to communication skills to be used with patients and relatives in nursing training could therefore be useful.

### Authors' Contributions

**BS:** Topic selection, writing the article, collecting and entering data.

**IA:** Writing the method and statistical evaluation.

**ASY:** Planning the study and preparing the manuscript.
